# Alginate Bead Biosystem for the Determination of Lactate in Sweat Using Image Analysis

**DOI:** 10.3390/bios11100379

**Published:** 2021-10-09

**Authors:** Sandra Garcia-Rey, Edilberto Ojeda, Udara Bimendra Gunatilake, Lourdes Basabe-Desmonts, Fernando Benito-Lopez

**Affiliations:** 1 Microfluidics Cluster UPV/EHU, Analytical Microsystems & Materials for Lab-on-a-Chip (AMMa-LOAC) Group, Analytical Chemistry Department, University of the Basque Country UPV/EHU, 48049 Leioa, Spain; sandra.garcia@ehu.eus (S.G.-R.); edilberto.ojeda@ehu.eus (E.O.); udarabimendra.kekulupolage@ehu.eus (U.B.G.); 2 Microfluidics Cluster UPV/EHU, BIOMICs Microfluidics Group, University of the Basque Country UPV/EHU, 01006 Vitoria-Gasteiz, Spain; 3 Bioaraba Health Research Institute, Microfluidics Cluster UPV/EHU, 01006 Vitoria-Gasteiz, Spain; 4 BCMaterials, Basque Center for Materials, Applications and Nanostructures, UPV/EHU Science Park, 48949 Leioa, Spain; 5 Basque Foundation of Science, IKERBASQUE, María Díaz Haroko Kalea, 3, 48013 Bilbao, Spain

**Keywords:** lactate, sweat, alginate bead, biosystem, colorimetric analysis

## Abstract

Lactate is present in sweat at high concentrations, being a metabolite of high interest in sport science and medicine. Therefore, the potential to determine lactate concentrations in physiological fluids, at the point of need with minimal invasiveness, is very valuable. In this work, the synthesis and performance of an alginate bead biosystem was investigated. Artificial sweat with different lactate concentrations was used as a proof of concept. The lactate detection was based on a colorimetric assay and an image analysis method using lactate oxidase, horseradish peroxidase and tetramethyl benzidine as the reaction mix. Lactate in artificial sweat was detected with a R² = 0.9907 in a linear range from 10 mM to 100 mM, with a limit of detection of 6.4 mM and a limit of quantification of 21.2 mM. Real sweat samples were used as a proof of concept to test the performance of the biosystem, obtaining a lactate concentration of 48 ± 3 mM. This novel sensing configuration, using alginate beads, gives a fast and reliable method for lactate sensing, which could be integrated into more complex analytical systems.

## 1. Introduction

Sweat is an aqueous solution that contains a great variety of compounds, such as NaCl, lactate [1], nitrogenous compounds (ammonia, urea and amino acids [2,3]), metal ions (e.g., zinc and iron [4,5]), heavy metals (e.g., arsenic, cadmium, lead and mercury [6]), immune biomarkers (e.g., IgG, IgD and interleukin-1α [7]), cortisol and stress biomarkers [8], lactoferrin [9] and xenobiotics (e.g., drugs of abuse [10] and ethanol [11,12]). The main function of sweat is thermoregulation, leading to heat dissipation by water evaporation [1] in response to an increase in body core temperature. Besides the regulation of body temperature, sweat also plays an important role in protecting, lubricating and waterproofing the skin. Moreover, sweat forms part of the immune system since it contains cytokines and other related molecules involved in the immune-mediated mechanisms that occur in the skin, being one of the first barriers during an immune response [13].

The high amount of physiological information contained in sweat, together with its accessibility in a non-invasive way, highlights the potential of sweat as an emerging alternative to standard blood analysis. Thereby, the analysis of sweat could provide an accurate insight into the physiological condition of the body (e.g., biomarker concentrations), but also a way of measuring body dehydration [14]. In fact, sweat is the diagnostic method for cystic fibrosis, a disease characterised by the presence of high levels of sodium and chloride in sweat [15,16].

In particular, lactate, which can be found in all physiological fluids, including sweat, is an important biomarker of fatigue and an indicative of restricted oxygen levels since it is a product of the anaerobic metabolism that takes place during the performance of intense exercise. Thereby, it is a metabolite of high interest in sport science and medicine, which can be used to keep track of the performance of athletes and to design a personalised training plan for each individual. In addition, it can be used as a diagnostic tool for disorders such as pressure ischemia, characterised by high levels of lactate [1]. However, based on previous literature, there is still some ambiguity regarding lactate concentration in sweat since it highly depends on, sweat rate, gender, age, environmental conditions, climate adaptation, fitness level and exercise intensity [1]. Moreover, sweat also shows variations in its composition depending on where it is collected from. Mickelsen O. et al. [17] measured sweat in five different body locations (torso, face, thigh, axilla and arm) and reported concentrations ranging from 16 mM on the face to 30 mM on the thigh. Despite the different lactate levels previously reported, its concentration in sweat always shows higher levels during the first measurements and begins to decrease when the sweat rate increases, which dilutes the lactate, until a plateau is reached [18]. The lack of standardisation underlines the importance of finding new methods for the determination of lactate levels in sweat, using sensing materials that can be coupled directly onto the skin to allow accurate and real-time measurements [19].

One of the possible biomaterials that could be employed to this end is alginate, a natural anionic polymer that can be found in brown algae and is composed of 1,4-linked α-*L*-guluronate and β-*D*-mannuronate residues arranged in linear copolymer blocks [20]. The biocompatibility, non-immunogenicity, porosity, viscosity and relatively low cost as well as gelation required to form dimension-controlled hydrogels of alginate make it an appropriate material to be used in a wide variety of fields, such as tissue engineering (e.g., scaffold for organ-on-a-chip, wound dressing), biomedical applications, drug delivery, food industry and environmental analysis (e.g., water analysis) [21,22,23].

Alginate hydrogels are formed by cross-linking the linear polysaccharide chains, thus creating a 3D network capable of retaining a high amount of water. The typical methods for alginate cross-linking are based on physical and chemical approaches: covalent cross-linking by the addition of a reagent that promotes covalent bonds between the alginate chains, thermal gelation based on temperature changes, cell cross-linking by adding cell adhesion ligands to the alginate and ionic cross-linking [21]. This last method is based on the combination of an alginate solution with divalent cations, such as Ca^2+^ and Ba^2+^, which bind the guluronate units allowing the cross-linking of the linear chains. These alginate hydrogels can be applied, for example, to alginate encapsulation of transplanted pancreatic islet cells in patients with type I diabetes as a way of protecting against a possible immune response of the host [24]. Despite its applications, alginate has just recently emerged as a potential technique for biosensing. In this regard, Márquez A. et al. [25] developed a point-of-care amperometric biosystem using a biocompatible alginate hydrogel matrix for glucose sensing in blood, and Zhuang, S. and Wang, J. [22] fabricated alginate beads modified with an indicator of Co^2+^ capable of effectively detecting Co^2+^ in aqueous solutions as an environmental analysis tool. Recently, our group has developed an alginate/titanium dioxide nanotubes nanocomposite for the detection of lactate and glucose [26].

The interest of using these types of biomarkers in sport science has grown considerably in recent years. Even though blood is still the gold standard method for analytical determinations in this field due to its inherent invasiveness, there is a high interest in developing non-invasive analyses, which could be achieved by sampling other biofluids, such as sweat. In particular, microfluidic technology is bringing sweat analysis in real-time to reality [27,28].

A wide range of biosensors have been developed in recent years [28,29,30]. Specifically, colorimetric detection, which allows immediate image analysis, is gaining interest for point of care as a fast, easy-to-use and versatile approach for sensing, due to the high quality of the cameras on the smartphones that are continuously appearing in the market. Moreover, the integration of wireless near-field communication (NFC) within microfluidics and wearable devices is enhancing the growth of the sector [31,32]. However, important aspects in the development of sweat analytical devices, such as efficient sweat collection, the sensitivity of the sensors and their correct operation in the specific circumstances of the analysis, still require optimisation. Furthermore, when the biosensor is formed of enzymes, one of the biggest challenges is maintaining its stability and activity for long periods of time in the final device. In these regards, the use of alginate-based hydrogels with integrated sensing moieties (enzyme assays) could overcome these limitations.

With the idea of being able to develop robust portable devices for the detection of lactate in sweat, in this work, we investigated the use of alginate beads as a biocompatible biosystem for the determination of lactate concentrations. We present the fabrication of alginate beads, the integration of a lactate enzymatic assay in the beads and the full characterisation of the assay for the determination of the lactate levels in both artificial sweat and real sweat samples using a “fit-to-purpose” colorimetric image analysis method. 

## 2. Materials and Methods

### 2.1. Artificial Sweat

Artificial sweat was made by mixing NaCl (99%, Sigma-Aldrich, Burlington, MA, USA), urea (Sigma-Aldrich, Burlington, MA, USA) and sodium *L*-lactate (Sigma-Aldrich, Burlington, MA, USA) at a concentration of 800 mM in distilled water and adjusted to a pH of 6.5. Solutions of lactate of 10, 20, 40, 60, 80 and 100 mM were prepared in artificial sweat at room temperature conditions. The solutions were stored at 2–8 °C until use. There was not an effect of varying the concentrations of NaCl and urea in the artificial sweat solution for the range of lactate concentrations of 10–100 mM, see Figure S1.

### 2.2. Solutions for the Synthesis of the Alginate Beads

The reaction mix for lactate sensing consists of lactate oxidase (LOD, 1KU, AGScientific, MA, USA), horseradish peroxidase (HRP, Sigma Aldrich, Burlington, MA, USA), tetramethyl benzidine (TMB Sigma Aldrich, Burlington, MA, USA) dissolved in dimethyl sulfoxide (DMSO, Sigma Aldrich, Burlington, MA, USA) and alginate (Sigma Aldrich, Burlington, MA, USA). LOD and HRP solutions were prepared in distilled water to a concentration of 0.40 and 0.05 mg mL^−1^, respectively. Twenty-four milligrams of TMB were dissolved in 2.25 mL of DMSO at 37 °C under continuous agitation for 2 h until its complete dissolution. An initial 2% alginate solution in distilled water was made, at room temperature and continuous agitation conditions, for 4 h until its complete dissolution. An initial solution of 2% alginate was prepared and diluted in distilled water to obtain solutions of 0.25, 0.50, 0.75, 1.00 and 1.50%. A CaCl_2_ (Sigma Aldrich, Burlington, MA, USA) solution was used for the formation of the alginate beads. It was prepared by mixing CaCl_2_ in distilled water to obtain a 400 mM solution (pH~7.5). All solutions were kept at 2–8 °C.

### 2.3. Fabrication and Characterisation of the Alginate Beads

For initial fabrication and characterisation, the alginate beads were made by mixing 10 µL of LOD, 10 µL of HRP and 5 µL of TMB solutions with 30 µL of alginate 1% (pH~8). The amount of each component varied during the optimisation experiments, as specified in Section 3.2 and Section 3.3. After mixing all the components together, 25 µL of the mix was taken with a pipette and dropped into the 400 mM CaCl_2_ solution. After 5 min, the formed alginate beads were washed with distilled water for 5 min and dried with filter paper, to remove any excess solution. The pH of the beads was measured from a water solution of 15 hydrogel scaffolds crushed, stored and settled in 1 mL distilled water for 24 h, obtaining a value of ~6.0. This pH should not affect the stability and performance of the enzymes. Then, the beads were placed in a 96-well white plate (Thermo Fisher Scientific, Waltham, MA, USA) and artificial sweat at different lactate concentrations was added for the analysis. Finally, images were taken at different times and analysed with ImageJ to measure the black and white index.

Bead fabrication and experiments were done at room temperature (25 ± 1 °C). The moisture conditions of the alginate hydrogel diminished the possible small temperature increments of the outside temperature by keeping the enzymes in an active state. Conventionally, evaporation of the water could have an effect, contracting the hydrogel bead, which could exert an external tension on the enzymes, leading to enzyme deactivation. In this regard, to avoid the contraction of the hydrogel, beads were stored in moisture conditions at 10–25 °C and used immediately after fabrication.

All the experiments were repeated by triplicate. The diameter of the alginate beads was measured 20 times.

Scanning electron microscopy (SEM) images of the freeze-dried alginate beads were recorded by Hitachi S-4800 (Hitachi, Tokyo, Japan) at an accelerating voltage of 5 kV.

### 2.4. Colorimetric Image Analysis

Different images of the alginate beads were taken during the experiments at 1, 2, 3, 4, 5, 6, 7, 8, 9, 10, 13, 16, 19, 25, 30, 35 and 50 min, using a 20 MP (megapixel) + 2 MP dual camera with an f/1.8 aperture (Huawei, Shenzhen, China) placed 25 cm over the sample. The same light conditions and camera settings were kept during all experiments using a photography chamber (MVpower Kit Photography Illumination Studio, Cube 80 × 80 × 80 cm^3^, 3× softbox 50 × 70 cm^2^, 3× light sources 135 W and 4× background colours, black, white, blue and red, Kissta). The images were later analysed using the image colour analysis software ImageJ. The original colour image was converted to an 8-bit grayscale and the black and white value (B/W value) of each bead was measured, scaling from 0–255 where black stands for 0 and white for 255.

## 3. Results and Discussion

### 3.1. Fabrication and Characterisation of the Alginate Beads

**Figure 1:**
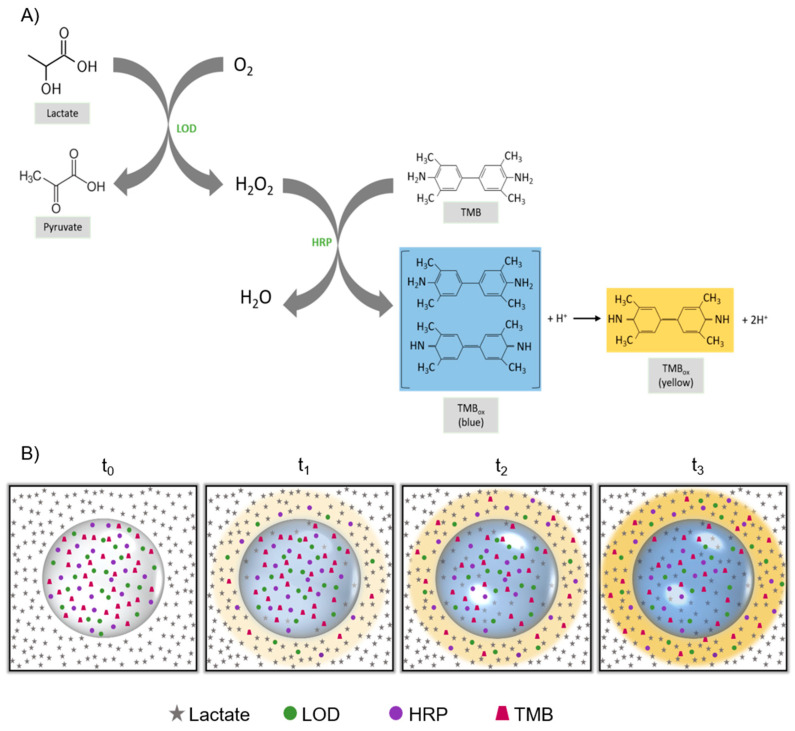
Enzymatic assay reaction when the components are integrated into the alginate bead sensor for lactate sensing. (**A**) Enzymatic cascade for lactate detection. The LOD oxidises the lactate present in sweat to pyruvate, yielding hydrogen peroxide, which is later used as the electron donor for the HRP. This enzyme performs the oxidation of TMB, yielding a blue-coloured product, which can be further oxidised in a two-electron oxidation as more hydrogen peroxide is generated, obtaining an orange/yellow-coloured product. (**B**) Schematic diagram of the alginate beads during lactate sensing over time (t_0_–t_3_).

This novel sensor approach is based on the reaction shown in Figure 1. Figure 1A represents a schematic diagram of the reaction that takes place inside the bead. First, the lactate present in the artificial sweat solution was oxidised to pyruvate by the LOD. The resulting H_2_O_2_ was then used by the HRP to oxidise the TMB. TMB is a diamine that, in the presence of an oxidising agent, is oxidized to a diimine and it can undergo either a one- or two-electron oxidation. The first coloured product, the resulting product of the one-electron oxidation, consists of a charge-transfer complex formed by the diamine, its oxidised diimine product and the radical cation, both species existing in equilibrium. This product absorbs light at 370 nm and 652 nm, providing to the material a characteristic blue colour. However, if this product is further oxidised, two-electron oxidation generates a diamine, absorbing visible light at 450 nm, yielding an orange/yellow-coloured product stable at acidic pHs [33].

After being added, the lactate solution covered the alginate bead while the enzymatic components of the mix remained inside the bead (Figure 1B, t_0_). Then, diffusion started in both directions, that is, lactate entered the bead while LOD, HRP and TMB slowly diffused outside, although this second process was observed to be much smaller due to the stabilisation of the assay components enacted upon the alginate matrix. As this happened, the TMB started becoming oxidised, yielding to the one-electron oxidation blue product inside the bead, giving the capsule a blue appearance (t_1_), which gradually developed from the edges of the bead towards the centre. On the other hand, outside the bead, the elevated concentration of lactate compared to the enzymatic components induced the complete oxidation of the TMB to its two-electron oxidation state, generating an orange/yellow solution surrounding the bead (t_1_). Then, diffusion through the alginate membrane continued in both directions, that is, lactate entered the bead while LOD, HRP and TMB diffused outside. As this happened, the TMB continued becoming oxidised, increasing the intensity of the blue colour of the bead. On the other hand, outside the bead the elevated concentration of lactate compared to the enzymatic components that leached out of the bead induced the complete oxidation of the TMB to its two-electron oxidation state. This yielded to an increase in the orange/yellow colour of the liquid surrounding the bead (t_2_). This condition (higher lactate concentration) remained the same during the length of the experiment, so the intensity of the orange/yellow-coloured liquid increased over time. The orange/yellow colour appeared first in the area closer to the bead, since it is there where the enzymatic assay reacts first with the lactate in the artificial sweat solution. As diffusion inside the bead occurred, the blue intensity increased inside the bead, whereas the yellow intensity in the surrounding liquid also increased due to the second oxidation of the TMB in the solution. This continued until an equilibrium was reached between the solutions outside and inside the bead (t_3_) and no further increase in the blue colour was appreciated in the bead. A video of the performance of a bead after the addition of 50 µL of solution of lactate 80 mM in artificial sweat is presented in Video S1.

**Figure 2:**
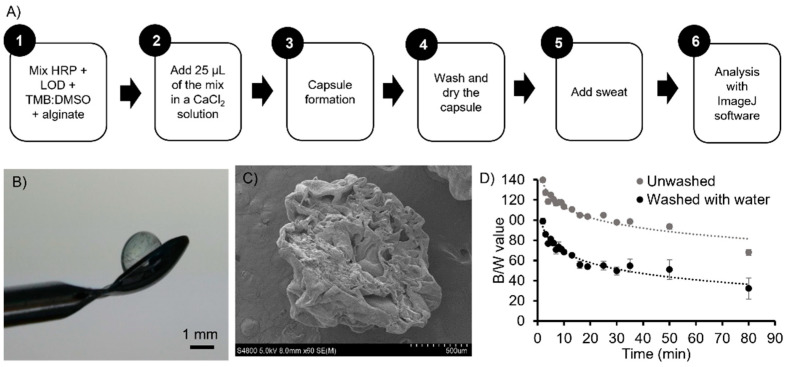
Alginate bead fabrication and characterisation. (**A**) Schematic diagram of the optimised fabrication protocol of the alginate beads. (**B**) Alginate bead after being washed with distilled water and dried. (**C**) SEM image of a cut alginate bead, showing its inner configuration. (**D**) Black and white values of the beads, obtained by image analysis, at different times after the addition of lactate 60 mM in artificial sweat. Values (grey dot) obtained from beads that were not washed with distilled water and dried. Values (black dot) obtained from beads washed with distilled water. Error bars correspond to mean values ± SD (n = 3).

Figure 2A shows the flow diagram for the fabrication of the alginate bead biosystems as explained in Section 2.3. Figure 2B shows an image of an alginate bead before adding the lactate solution. The alginate beads were clear and uniform, and presented a well-defined spherical shape with a diameter of 2.3 ± 0.2 mm (n = 20). This variability was due to human handling and to the viscosity of the alginate. In Figure 2C an SEM image of the alginate bead cut in half is presented.

As a first characterisation, the colour of the beads, after adding artificial sweat containing lactate (60 mM), was analysed before and after the washing/drying step. The obtained B/W values are shown in Figure 2D; both assays followed the same tendency, but the unwashed beads had higher B/W values and thus a lighter blue colour, see Figure S2. In both cases, the formation of an orange/yellow colour in the solution, surrounding the bead, was observed. In the unwashed beads experiments, the orange/yellow colour of the surrounding solution was more intense than in the washed/dried beads, and rapidly covered the full solution in the well. This was due to the excess of mix solution on the surface of the bead that diffused into the artificial sweat solution. Additionally, the intensity of the blue colour over time was not as pronounced as in the washed/dried beads. This effect was explained by looking at the porosity of the alginate. In the case of the unwashed beads, there was an excess of reagents (enzymes and TMB) in the solution, coming from the surface of the bead. Therefore, the reaction immediately started in the outside of the bead, since lactate was in excess in the solution, proceeding towards the two-electron oxidation of the TMB (orange/yellow product). By the time the diffusion process became noticeable, since lactate diffusion through the bead is a slower process than the reaction in the solution, there was little lactate left available surrounding the bead. Therefore, the observed blue colour of the bead was not very intense—see Figure S2—even at long reaction times, up to 35 min.

By washing the beads before the addition of the artificial sweat solutions (Figure 2D), the excess of enzymes and TMB from the surface of the beads was removed, increasing the sensitivity of the colorimetric analysis. Thus, when artificial sweat was added, lactate did not react in the solution, and had time to diffuse inside the bead in order to perform the reaction. This allowed the bead to increase its blue intensity overtime until the system reached the equilibrium.

PBS 1% was also tested as a solution to wash the beads with instead of distilled water, as it is a biological buffer. However, the obtained B/W values of the beads were not as homogenous as the ones obtained when washing with distilled water. Moreover, it was observed that TMB precipitates inside the beads (Figure S3), which affected the colorimetric analysis. From this point onward, all experiments were performed using washed/dried beads.

### 3.2. Optimisation of the Mix Reagents

Alginate concentrations of 0.5, 0.75, 1, 1.5 and 2% were tested for the synthesis of the beads. No alginate beads with an alginate concentration lower than 0.5% were tested because the obtained hydrogel would be too weak to form a bead (Figure S4). Likewise, no beads with an alginate concentration higher than 2% were tested due to the high viscosity of the alginate, which hindered its manipulation and the homogeneity of the generated beads.

**Figure 3:**
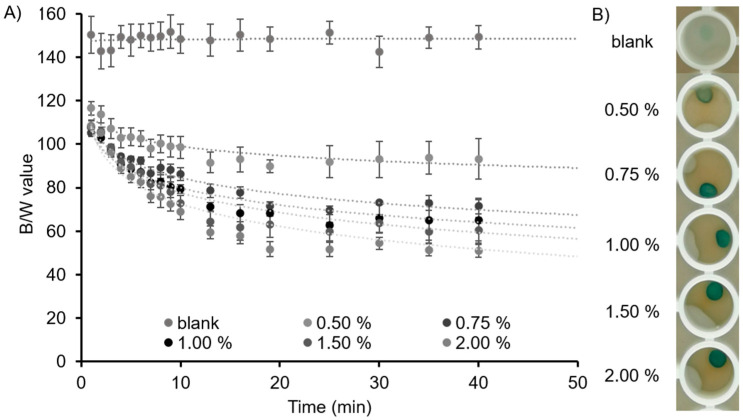
Optimisation of the alginate concentration for the fabrication of alginate beads. (**A**) Black and white values of the beads with alginate 0.5, 0.75, 1, 1.5 and 2%, after adding an artificial sweat solution with a lactate concentration of 60 mM at different times. The blank was an alginate bead, 1.5%, after adding an artificial sweat solution without lactate, as a reference. (**B**) Pictures of beads with alginate concentrations of 0.5, 0.75, 1, 1.5 and 2% taken at 13 min, when the reaction reached a plateau. Error bars correspond to mean values ± SD (n = 3).

The B/W values of the different alginate beads were obtained when immersing the beads, up to 40 min, in an artificial sweat solution with a lactate concentration of 60 mM, Figure 3A. As can be appreciated, the B/W values of the blank beads barely changed throughout the experiment, since the solution did not contain any lactate. When lactate was added to the beads, the obtained B/W values were higher and there was a lighter blue colour for the beads with a lower % of alginate. This behaviour was observed throughout all the times investigated. As an example, Figure 3B shows how the blue colour of the beads increased with the alginate concentration at time 13 min.

The reason behind this phenomenon lies in the concentration of alginate in the bead, which generates thicker beads when the percentage of alginate is increased. In the beads made with higher alginate concentrations, lactate diffused inside slowly, and thus lead to the generation of a lower amount of H_2_O_2_, promoting just the one-electron oxidation of TMB. Thus, the beads with higher alginate concentrations appeared as a darker blue, with subsequent lower B/W values.

Alginate 0.5% and 0.75% beads were fabricated, but their shape was not homogeneous, and they were not robust enough to be used as material in a biosystem. On the other hand, beads made with alginate 2% were robust and the variation in the B/W value, after the assay performance, was small (±3%). However, alginate at this concentration is very viscous, which made its manipulation tedious. Therefore, this percentage of alginate was not used for further developments of the material. Thereby, either alginate 1% or alginate 1.5% were the concentrations that worked the best when preparing alginate beads since they combined an easy manipulation of the alginate, homogenous colour distribution when lactate was added and a good formation as well as durability of the beads. For further experiments, alginate 1.5% was chosen due to its slightly higher robustness. In general, a plateau in the B/W values from minute 13 was observed for all the alginate concentrations, which represents the point where the assay reached the equilibrium.

The calibration curve for the lactate assay in the beads made with alginate in the range from 0.5% to 2% at 13 min is shown in Figure S5. Results demonstrated that, despite alginate 1.5% being chosen for the fabrication of the beads in this investigation, other alginate concentrations could be used for the fabrication of the beads, since the B/W values obtained were consistent and reliable, opening the detection capabilities and versatility of the proposed biosensing material. Finally, the optimisation of LOD, HRP and TMB concentrations is explained in Figure S6.

### 3.3. Calibration Curve for Lactate in Artificial Sweat

**Figure 4:**
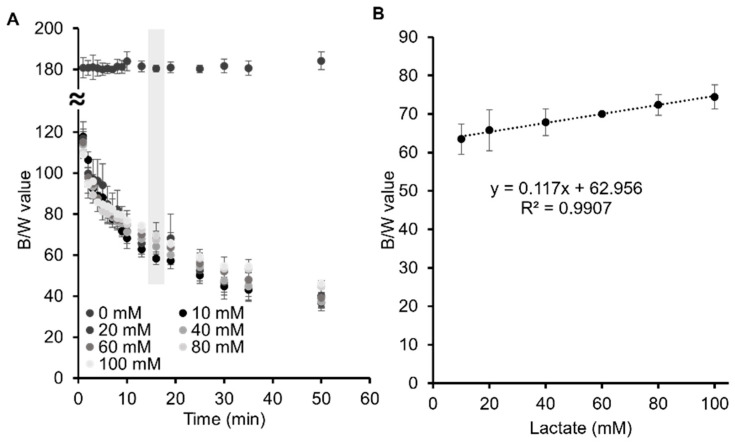
(**A**) B/W values of alginate beads tested with artificial sweat containing 0, 10, 20, 40, 60, 80 and 100 mM concentration of lactate. The shadowed values indicate the time when the plateau is reached, 13 min. (**B**) External calibration line at 13 min for the determination of lactate in the range of 10 mM to 100 mM in artificial sweat. The calibration curve was defined by the equation y = 0.117x + 62.956, with a R² = 0.9907. Error bars correspond to mean values ± SD (n = 3).

Once the concentration of alginate and the mix of enzymes and TMB were optimised for the fabrication of the bead biosystem, different artificial sweat solutions with lactate concentrations of 0, 10, 20, 40, 60, 80 and 100 mM were tested, as the lactate concentration in sweat has been reported to be in the range of 10–70 mM [34,35,36]. As shown in Figure 4A, the colour of the beads barely changed when lactate is not present in the solution. However, the B/W values increased as the lactate concentration increased, the alginate beads with lactate 100 mM solution being the ones with the highest B/W values, while the alginate beads with lactate 10 mM solution showed the lowest B/W values. This means that, as the lactate concentration increases, the blue intensity of the beads decreases, which is due to the second-electron oxidation of TMB that takes place inside the bead when lactate is absorbed inside of the beads. As demonstrated in Section 3.2, the more lactate inside the alginate beads, the more the reaction will develop towards the complete second-electron oxidation of the TMB, and thus the formation of the orange/yellow product. Consequently, the alginate beads with lactate 100 mM showed a lighter blue intensity, which gradually increased as the lactate concentration of the solution surrounding the beads decreased.

At minute thirteen the reaction reached a plateau because of the saturation of the enzymes and the lack of TMB available. Afterwards, the B/W values were stable within the error until minute 35. Since all the lactate concentrations presented distinguishable B/W values at this point, the values were used to build an external calibration curve, Figure 4B. Lactate concentrations were determined in a linear calibration range of 10 mM to 100 mM, which is compatible with the sweat lactate values obtained in the literature, as referenced before. A limit of detection of 6.4 mM and a limit of quantification of 21.2 mM were obtained (the limit of detection was calculated as LoD = 3 SD/k and the limit of quantification as LoQ = 10 SD/k, where SD is the standard deviation of the blank and k is the slope).

In contrast to blood, there is not an established range of lactate concentration in sweat since it depends on fitness level, age, sex, gender and the place where the sweat is collected from [1,18,37]. Therefore, a wide variety of lactate concentrations have been reported in sweat. Promphet N. et al. [38] were able to measure lactate levels of 13, 60 and 64 mM in three volunteers after 30 min of jogging using a non-invasive textile-based colorimetric sensor. On the other hand, Kim, S. B. et al. [31] detected an average lactate concentration of 14 mM after exposing three subjects to cycling on a stationary bike with increasing resistance. In later research, the same group developed a microfluidic device for the colorimetric detection of lactate in sweat that detected levels between 15 mM to 22 mM in healthy subjects after cycling exercises [39]. Therefore, according to the results found in the literature, our alginate-based sensor can be employed for lactate detection in sweat samples, since its detection range sits within the human physiological lactate sweat range.

To verify the potential of using alginate beads as biosystems for lactate determination using image analysis, two samples of artificial sweat were prepared with unknown lactate concentrations. Alginate beads were immersed in the two samples and the B/W values were obtained at 13 min. The values were plotted in the external calibration line, obtaining values of 18 ± 2 and 50 ± 7 mM, respectively (error bars correspond to mean values ± SD, n = 3). The real values of those two solutions were 20 mM and 45 mM, respectively. These results demonstrated that accurate and reliable measurements of lactate concentrations in artificial sweat could be obtained using an enzymatic alginate bead biosystem.

### 3.4. Validation of the Biosystem with Real Sweat Samples

For the validation of the biosystem, real sweat was collected from a healthy female (age 28) after 45 min of indoor cycling. A total volume of 50 µL was collected from the forehead and stored into an Eppendorf tube until use. In order to have enough volume to run three assays, the sample was diluted three times in distilled water. Of the sample, 50 µL was added to the bead and the lactate concentration was measured after 13 min by plotting the measured B/W value into the calibration curve obtained before. A lactate concentration of 48 ± 3 mM (n = 3) was obtained for sweat. The concentration of the sample was also measured using a commercially available device (Lactate Plus, Nova Biomedical, Waltham, MA, USA) for the determination of lactate levels, obtaining a concentration of 56 ± 1 mM (n = 3).

These results demonstrate that the biosystem developed in this work can, indeed, be used for lactate determination in real sweat samples within the physiological range, after colorimetric analysis of the alginate beads. However, since lactate levels during exercise vary between individuals and over exercise time, no analytical conclusions can be obtained from this result yet. In order to get them, continuous tracking of sweat lactate levels would need to be done by integrating the biomaterial within a wearable device. The implementation of these type of devices has not been solved yet, since it is difficult to obtain reliable sweat samples without compromising sweat analyte concentrations and avoiding contamination [28,40,41,42]. Moreover, this biosystem could also be applied in the medical field for the detection of diseases such as cystic fibrosis. However, more research needs to be done regarding the possible relation between sweat and blood lactate levels.

## 4. Conclusions

In this work, we have demonstrated and discussed the synthesis and application of alginate beads as novel biocompatible materials for lactate sensing in artificial and real sweat. Lactate concentrations were determined using a colorimetric alginate-based biosystem integrated with LOD, HRP and TMB in 1.5% alginate beads. This method permitted the detection of up to 6.4 mM concentration of lactate and the quantification of up to 21.2 mM of lactate in artificial sweat. Moreover, as a proof of concept, this newly developed biosystem was tested with real sweat samples, demonstrating its possible applicability in real-case scenarios.

Compared to standard blood analysis, this new technology provides the possibility of measuring lactate in a non-invasive biofluid, such as sweat. Moreover, since the enzymatic components are immobilised in a hydrogel, the reaction takes place inside the bead, providing higher sensitivity for the colorimetric analysis using image analysis. This, together with the ease of manipulation of alginate beads and lower detection times of up to 15 min, will allow the integration of this biosystem into a microfluidic platform and its application to monitor lactate levels at the point and place of need.

The possibility of measuring lactate levels in sweat in its physiological range using alginate bead biosystems and image analysis opens up many options in the development of easy-to-use biosensors for wearable technology and wearable applications. Since alginate is a biocompatible, non-immunogenic and porous hydrogel that can be fabricated at low cost, its use as biosensing material will open new avenues in sensor devices. With lactate being a biomarker of anaerobic metabolism, the work developed here opens a door to a new way of lactate determination by using alginate, with applications in sports science and medicine.

## Data Availability

Data will be available in Zenodo: https://zenodo.org/communities/mami-h2020 accessed on 19 August 2021.

## Supplementary Materials

The following are available online at www.mdpi.com/article/10.3390/bios11100379/s1, Figure S1: Effect of the concentration of NaCl and urea in the artificial sweat solution for lactate sensing, Figure S2: Unwashed beads and beads washed with distilled water at 4, 16 and 35 min after the addition of lactate, Figure S3: Alginate beads washed with PBS 1%, Figure S4: Alginate bead fabricated with alginate 0.5% at 8, 25, and 80 min, Figure S5: Calibration curve of alginate beads ranging from 0.5 to 2% at 13 min, Figure S6: Optimisation of the amounts of LOD, HRP and TMB solutions inside the bead for lactate sensing, Video S1: Performance of a bead after addition of 50 µL of solution of lactate 80 mM in artificial sweat. The speed of the video was increased to 60× for visualisation.
