# Supplementary material for: Alginate Bead Biosystem for the Determination of Lactate in Sweat Using Image Analysis
Source: Biosensors (Basel). 2021 Oct 9;11(10):379. doi: 10.3390/bios11100379 (PMC8534122; doi:10.3390/bios11100379)
Supplement: Supplementary file 1 [file biosensors-11-00379-s001.zip › biosensors-1367162-supplementary.pdf]

*SUPPLEMENTARY INFORMATION*

# Alginate Bead Biosystem for the Determination of Lactate in Sweat Using Image Analysis

**Sandra Garcia-Rey [1,2], Edilberto Ojeda [1,2], Udara Bimendra Gunatilake [1,2], Lourdes Basabe-Desmonts [2,3,4,5,*] and Fernando Benito-Lopez [1,2,3,*]**

[1] Microfluidics Cluster UPV/EHU, Analytical Microsystems & Materials for Lab-on-a-Chip (AMMa-LOAC) Group, Analytical Chemistry Department, University of the Basque Country UPV/EHU, Leioa, 48049, Spain; sandra.garcia@ehu.eus (S.G.-R.); edilberto.ojeda@ehu.eus (E.O.); udarabimendra.kekulupolage@ehu.eus (U.B.G.)

[2] Microfluidics Cluster UPV/EHU, BIOMICs microfluidics Group, University of the Basque Country UPV/EHU, Vitoria-Gasteiz, 01006, Spain.

[3] Bioaraba Health Research Institute, Microfluidics Cluster UPV/EHU, Vitoria-Gasteiz, 01006, Spain

[4] BCMaterials, Basque Center for Materials, Applications and Nanostructures, UPV/EHU Science Park, Leioa, 48949, Spain

[5] Basque Foundation of Science, IKERBASQUE, María Díaz Haroko Kalea, 3, Bilbao, 48013, Spain

* Correspondence: lourdes.basabe@ehu.eus (L.B.-D.); fernando.benito@ehu.eus (F.B.-L.)

**Supporting Information 1 (S1).**

**Supporting Information 2 (S2).**

**Supporting Information 3 (S3).**

**Supporting Information 4 (S4).**

**Supporting Information 5 (S5).**

**Supporting Information 6 (S6).**

**Supporting Information – Video 1**

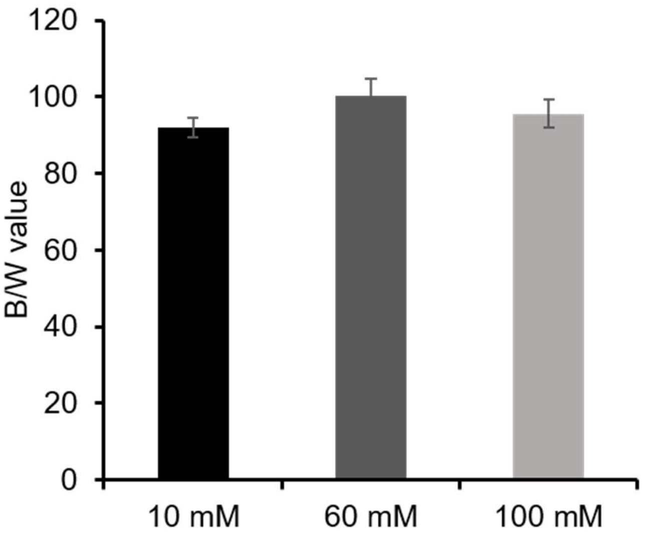


**Figure S1.** Effect of the concentration of NaCl and urea in the artificial sweat solution for lactate sensing. NaCl and urea 10, 60 and 100 mM were tested with lactate at 60 mM. The B/W value for lactate 60 mM falls within the range in the three situations tested, with an average value of 95 ± 4. Error bars correspond to mean values ± SD (n = 3).

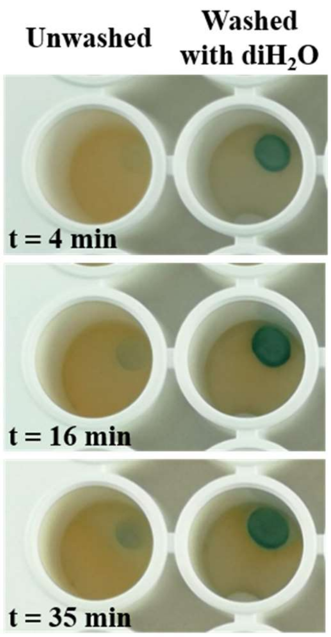


**Figure S2..** Unwashed beads (**A**) and beads washed with distilled water (**B**) at 4, 16 and 35 min after the addition of lactate. Unlike in the washed beads, the reaction takes place mainly in the surrounding medium when using the unwashed beads because of the remaining components of the mix on their surface, giving rise to an orange/yellow colour. However, after washing the beads, instead of reacting on the surface, the lactate enters the beads, where the reaction starts.

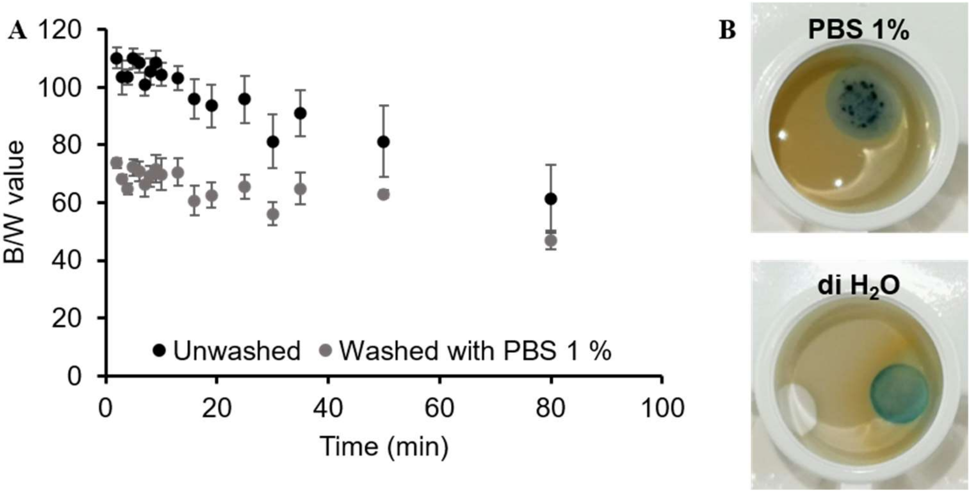


**Figure S3.** Alginate beads washed with PBS 1%. (**A**) Black and white (B/W) values of the unwashed beads (black) and the beads washed with PBS 1% (grey), for 80 min. (**B**) Alginate bead washed with PBS 1% and with distilled water (di $H_2O$) at 13 min, after adding lactate 60 mM in artificial sweat. As it is shown in the image, when washing with PBS, the TMB aggregated inside the bead, which difficulties the colorimetric analysis.

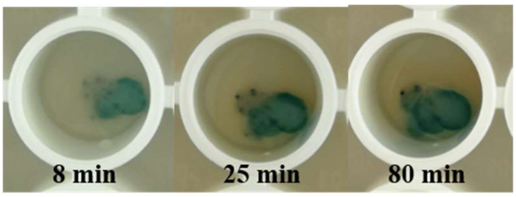


**Figure S4.** Alginate bead fabricated with alginate 0.5% at 8, 25, and 80 min. The obtained hydrogels were too weak to maintain the spherical shape. Thus, part of the enzymatic mix spread in the surrounding liquid. No alginate concentration under 0.5% was tested for the fabrication of the beads.

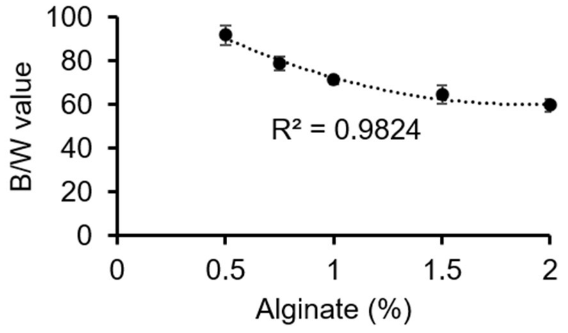


**Figure S5.** Calibration curve of alginate beads ranging from 0.5 to 2% at 13 min. The calibration curve is defined by the equation y = 15.947 × 2 − 59.766x + 116.07, with a $R^2$ = 0.9824. Error bars correspond to mean values ± SD (n = 3).

**Optimisation of the amount of LOD, HRP and TMB**

For the optimisation of the LOD, HRP and TMB four different reaction mixes were prepared. Mix-A consists of 2.5 µL of LOD, 2.5 µL of HRP and 1 µL of TMB solutions with 30 µL of alginate 1.5%; mix-B consists of 20 µL of LOD, 20 µL of HRP and 10 µL of TMB solutions with 30 µL of alginate 1.5%; mix-C consists of 10 µL of LOD, 10 µL of HRP and 5 µL of TMB solutions with 30 µL of alginate 1.5% and mix-D consists of 5 µL of LOD, 5 µL of HRP and 3 µL of TMB solutions with 30 µL of alginate 1.5%. Alginate beads were fabricated as described in Section 2.3 using mix-A, mix-B, mix-C and mix-D and an artificial sweat, lactate 60 mM, solution was added to the beads to perform the assay.

The concentration of LOD, HRP and TMB was optimised to be able to detect lactate in artificial sweat with measurable colour intensity. In order to investigate the most appropriate volume of each of the reactant solutions of the biosensor, four different mixtures were investigated in the 1.5% alginate beads, see experimental section too. All the beads were fabricated with the same amount of alginate and the same lactate concentration was added to perform the assay in the four type of beads. As in previous experiments, the same proportion between each enzyme and TMB was kept, 2:1, with a slightly variation of 0.25 (2:1.25) of TMB concentration in one of the bead types.

The B/W values over time, SI-Figure 6, showed the same tendency for the four solutions investigated (mix-A-D) with a plateau at 13 min. However, Mix-A and -B showed similar B/W values, within the error, whereas mix-C and -D showed lower B/W values, also similar to each other within the error. In the case of mix-A, the amount of the mix components was not enough to provide sufficient dark blue colour to the bead during the performance of the assay. In mix-B, the volume of mix component solutions and the volume of alginate solution used to form the bead were the same (15 µL), which generated weaker cross-linked alginate beads. Therefore, the beads had low stability and very high porosity, allowing the components from the enzymatic assay to quickly diffuse outside the bead and thus, promoting that the reaction takes place in the liquid surrounding the bead. Moreover, due to the high lactate concentration outside the bead, the leached TMB completely oxidised to the second-electron oxidation, generating a light-coloured bead (less blue and more orange/yellow colour). Moreover, due to the lack of robustness, the bead barely maintained its spherical shape over the experiment.

The B/W values for mix-C and -D were similar, obtaining beads with darker blue colour compared to the other mixtures. Although mix-C and -D had proper reagent concentrations to get a functional alginate bead, mix-D contained half of the amount of LOD, HRP and TMB reagents, reducing the waste of reagents. Therefore, mix-D was chosen as the solution for the fabrication of the bead biosensor from now on.. Nevertheless, it should be considered that other enzymes ratios or H2O2/TMB rations could provide better results or performances. A compromise about the performance of the assay and the robustness of the alginate bead, needs to be considered as well for the fabrication of this biosystem.

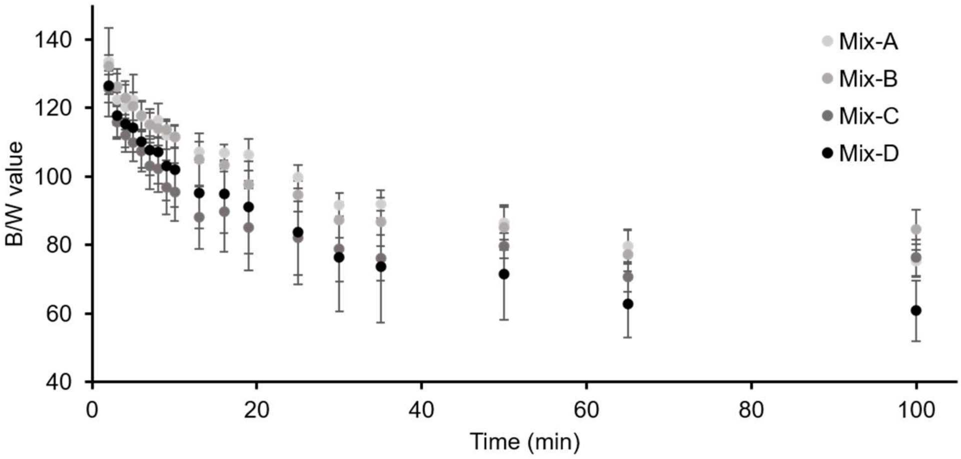


**Figure S6.** B/W values of the four different mixes used for the optimisation of the amounts of LOD, HRP and TMB solutions inside the bead for lactate sensing.

**Scheme S1.** A video of the performance of a bead after addition of 50 μL of solution of lactate 80 mM in artificial sweat. The speed of the video was increased to 60X for visualisation.
